# Basilar apex artery aneurysm clipping: how I do it

**DOI:** 10.1007/s00701-025-06687-1

**Published:** 2025-10-06

**Authors:** Paolo Palmisciano, Sudhakar Vadivelu, Norberto Andaluz, Mario Zuccarello

**Affiliations:** 1 Department of Neurosurgery, University of California Davis, Sacramento, CA USA; 2 Department of Pediatric Neurosurgery, Cincinnati Children’s Hospital Medical Center, Cincinnati, OH USA; 3 Department of Neurosurgery, University of Cincinnati College of Medicine, Cincinnati, OH USA

**Keywords:** Anatomy, Basilar artery aneurysm, Clipping, Neuromonitoring, Vascular neurosurgery, Vasospasm

## Abstract

Approximately 5–8% of intracranial aneurysms arise from the basilar artery apex (BAA). Given the basilar artery role in supplying the brainstem and the posterior cerebral circulation, the rates of morbidity and mortality are especially high in case of rupture. Although endovascular occlusion techniques are overall favored, thus leading to decreasing surgical expertise, complex BAA aneurysm may still require treatment with microsurgical occlusion. We describe our institutional perioperative protocol in the management of BAA aneurysms, focusing on surgical anatomy, approach selection, and intraoperative nuances.

**Supplementary Information:**

The online version contains supplementary material available at 10.1007/s00701-025-06687-1.

## Introduction

Aneurysms arising from the BA apex (BAA) comprise approximately 5–8% of all intracranial aneurysms, yet, when ruptured, harbor high rates of morbidity and mortality [1, 3]. Although endovascular techniques are the current standard, complex BAA aneurysms may incorporate factors that favor clipping: large/giant size, wide neck, partial thrombosis, involvement of the branches/perforators on the aneurysmal dome [1, 10]. We describe our institutional surgical management of BAA aneurysms.

## Surgical anatomy

The BAA is located anteriorly to the midbrain and posteriorly to the mammillary bodies within the interpeduncular cistern; it bifurcates proximally into the bilateral superior cerebellar arteries (SCA) and distally into the bilateral PCAs-P1 segments, with oculomotor nerves in between. The BAA lies posteriorly and superiorly to the dorsum sellae and posterior clinoid processes (PCPs; high bifurcation), medially to the petrous apex, and inferiorly to the tentorium [6]. Less commonly, the BAA lies below the PCPs (low-bifurcation) [6]. Although the posterior thalamo-perforating arteries (PTPAs) typically arise from the P1 segments within 0.4–4.7 mm from the BAA, they may arise directly from the BAA, posing higher surgical risks [7]. Injury to the PTPA may lead to coma, thalamic infarcts, or death.

The subarachnoid cisterns provide natural corridors to the BAA: 1) interpeduncular cistern, housing the BAA and perforators; 2) chiasmatic and carotid cisterns, allowing exposure of proximal BA; 3) the crural/ambient cisterns, allowing exposure of P1 segments. The Liliequist membrane, a two-layered arachnoid structure, separates the chiasmatic/carotid cisterns from the interpeduncular and prepontine cisterns; its careful opening is critical to access the BAA safely while minimizing retraction.

Rare BAA variants have been reported, highlighting the importance of obtaining preoperative vascular imaging. Cadaveric studies have described: 1) BAA fenestration in 0.9–2.1% of cases; 2) BAA trifurcation, giving origin to a third fetal-type PCA or accessory PCA; 3) asymmetric PCA origin, with one dominant and one hypoplastic PCA; 4) common trunk including PCAs and SCAs origins. The presence of persistent carotid-basilar anastomoses needs to be considered, including persistent primitive trigeminal (0.5–0.7%), hypoglossal (0.027–0.29%), otic, and proatlantal arteries [4].

Figures 1, 2 and 3.

**Fig. 1:**
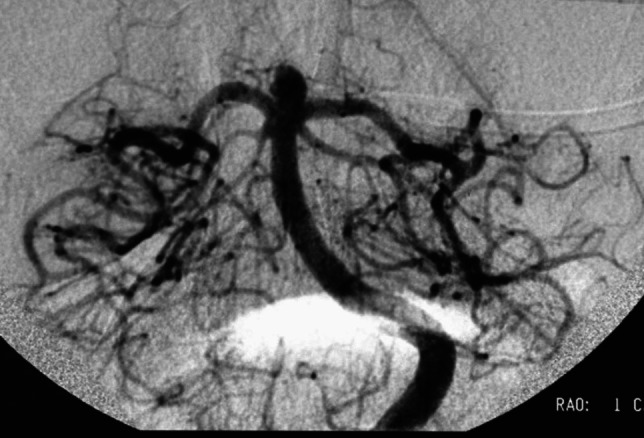
Digital subtraction angiogram showing unruptured basilar tip aneurysm projected supero-anteriorly

**Fig. 2:**
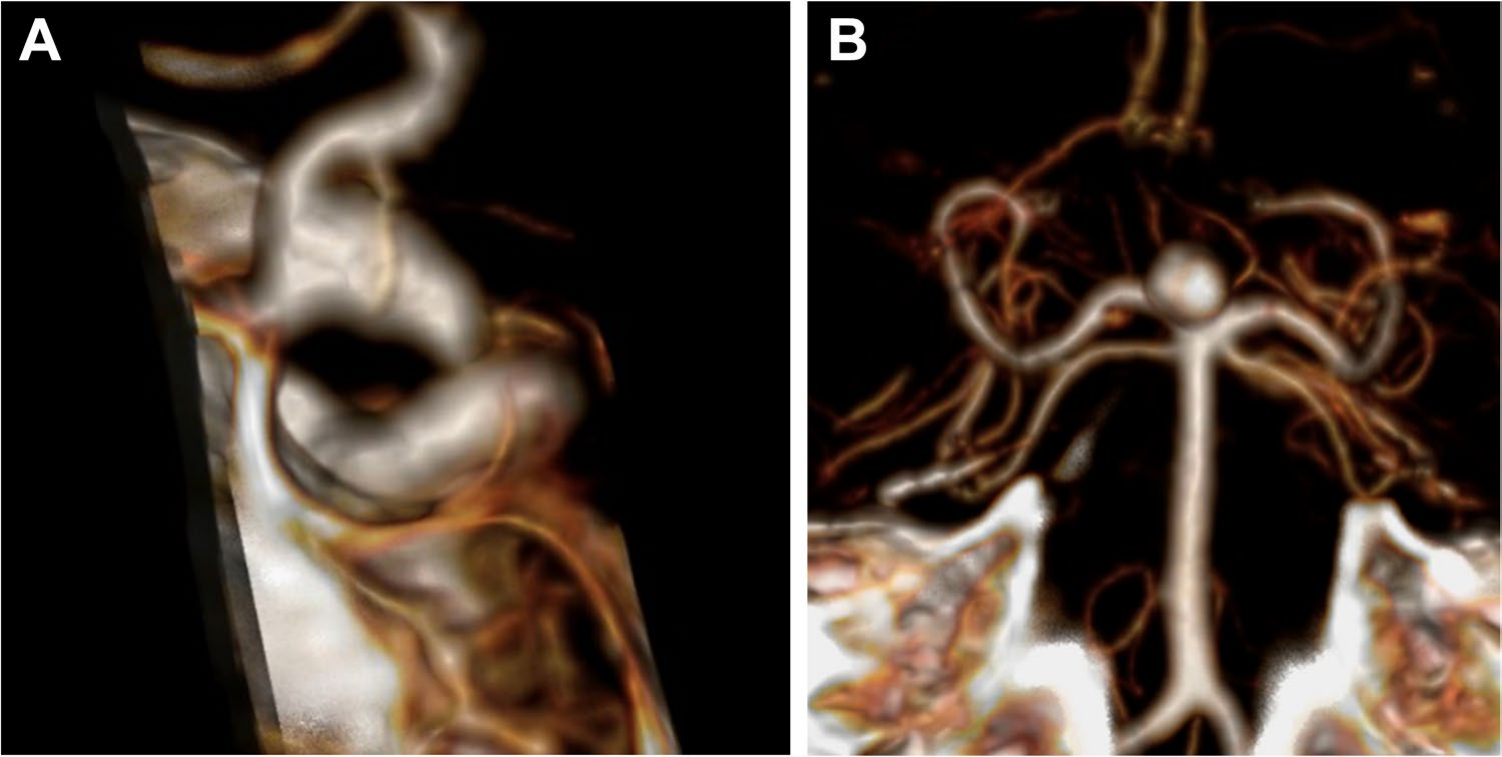
CT 3D reconstruction showing the basilar tip unruptured aneurysm in (**A**). sagittal and (**B**). coronal views

**Fig. 3:**
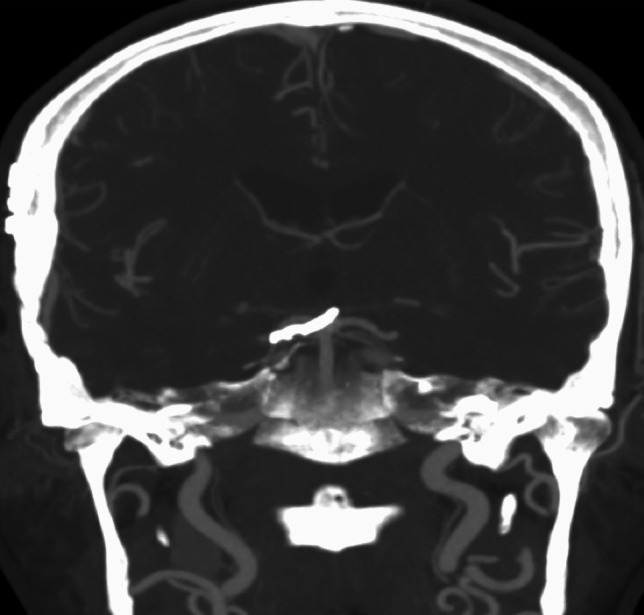
Postoperative CT angiogram showing complete clip occlusion of the basilar tip aneurysm

**Video 1**. Description of right frontotemporo-orbitozygomatic approach with access and clipping of an unruptured basilar tip aneurysm through the optic-carotid window aided by PAVEL approach.

## Surgical technique

### Preoperative assessment

CT angiography and/or digital subtraction angiography (DSA) are necessary to characterize the aneurysm morphology and the related vascular structures, allowing optimal approach planning. For unruptured aneurysms, MRI may evaluate their relationships with surrounding non-vascular structures.

Pre-operative imaging is necessary for evaluating:

1. Aneurysm projection, height, width, branching vessels, and adherence to surrounding structures.
2. Size of the aneurysm neck.
3. Relationship of the sac with perforators and the PCAs.
4. Distance from the sagittal midline and the clivus.
5. Level of bifurcation riding related to the clivus and PCP, based on the “clival zones” [1]:
  - *Zone 0a*: supraclinoidal, < 10 mm from the PCP (low-riding BAA aneurysms), best approached through an anterolateral approach (pterional craniotomy and variants);
  - *Zone 0b*: high basilar, > 10 mm from the PCP (low-riding BAA aneurysms), best accessed through a frontotemporo-orbitozygomatic (FTOZ) approach;
  - *Zone Ia*: between the PCP and the sellar floor, best reached through a subtemporal approach ± zygomatic osteotomy;
  - *Zone Ib*: between the sellar floor and the internal auditory canal (IAC), best targeted through an anterior petrosectomy (Kawase) approach.
6. Internal carotid artery (ICA) length and direction.
7. Presence of other aneurysms.
8. Presence of subarachnoid hemorrhage and temporal lobe swelling.

We will discuss the management of “*zone 0 (a/b)*” BAA aneurysms, which are the most treated.

### Pre-procedure plan

1. Right-side favored for right-handed surgeons.
2. Supine positioning.
3. Head elevated 10–20° above level of heart and turned 30–45° towards the contralateral side to provide better access to the Sylvian fissure.
4. Use the intraoperative neuromonitoring.
5. Maximal cerebrospinal fluid (CSF) release by placing an external ventricular drain or lumbar drain.
6. Intraoperative mannitol is not routinely used.

### Windows to access the BAA

After completion of the craniotomy and wide sylvian fissure dissection, four routes can be approached:

- Lateral retro-carotid (preferred): corridor through the carotid-oculomotor window limited by the anterior clinoid process (ACP), supraclinoid ICA, oculomotor nerve, and the PCP. This corridor can be expanded by resecting the medial temporal pole (“PAVEL” approach [5]; limited to 5–10 mm anteriorly, in case of high-riding or concealed aneurysms [9]), drilling the ACP and/or PCP, and mobilizing the ICA and the oculomotor nerve (transcavernous approach).
- Optico-carotid: triangle between the ICA, the optic nerve, and the ACA-A1 segment. Very narrow corridor that requires drilling of the ACP, unroofing of the optic canal, and possible drilling of the PCP. In the presence of a good anterior communicating complex, the A1 segment may be transected to allow greater access.
- Medial retrocarotid: corridor between the ICA and the posterior communicating artery (PCoA). Recommended with high-riding BAA or too short P1 segment. This space is traversed by the PCoA perforators.
- Supracarotid/supra-bifurcation: corridor above the ICA. Recommended when ICA atherosclerosis prevents its mobilization, or the ICA is too short, or the optico-carotid window is too narrow. The optic tract and lenticulostriate arteries are usually in the way.

### Our technique

A standard pterional craniotomy with wide sphenoid wing flattening is preferred for *zone 0a* aneurysm, while an FTOZ approach (one-piece at our institution [2]) is more suitable for zone 0b aneurysm. An FTOZ approach may be used for *zone 0a* aneurysms in patients with subarachnoid hemorrhage and swollen brain. Improved management of temporalis muscle has reduced the need for zygomatic arch detachment, requiring only a superolateral orbitotomy.

1. Anterior clinoidectomy:
  - Elevate the skull base dura posteriorly to expose the optic foramen.
  - Elevate the temporal dura anteriorly along the superior orbital fissure.
  - Cut the meningo-orbital ligament.
  - Dissect the ACP from all dural adhesions and drill with diamond burr under irrigation.
  - Unroof the optic canal.
2. Mobilization of the ICA:
  - Dolenc Y-shaped incision with limbs along the sylvian, subfrontal, and temporal fissures.
  - Expose the dural ring.
  - Divide the falciform ligament.
  - Cut the distal dural ring circumferentially around the ICA.
  - Mobilize the ICA from the carotid groove and retract it medially.
3. Posterior clinoidectomy:
  - Incise the dura overlying the PCP longitudinally along its posterior aspect to expose the PCP
  - Continue cutting the dural flap towards the cavernous sinus
  - Cut the posterior flap with micro-scissors
  - Drill the PCP and the dorsum sellae with diamond burr (the dural flap protects the cavernous sinus)

These maneuvers increase the exposure of the basilar trunk through the carotid-oculomotor window by 70% for proximal control of the BA [1].

### Clip selection

- Anteriorly projecting aneurysms: straight clip parallel to the base of the aneurysm; no perforators are at risk.
- Superiorly projecting aneurysms: straight fenestrated clip with the ipsilateral PCA into the fenestration, and the tips of the clip stop at the origin of the contralateral PCA; perforators need to be identified and dissected free prior to clip application.

Reduce clip repositioning in the event of aneurysm sac thrombosis. If feasible, for large or giant aneurysms, tandem clip application may be favored to better manage neck visualization and clipping while preserving perforator branches.

## Indications

- Superior/anterior projecting aneurysms (ruptured and unruptured)
- Poor endovascular access
  - Dome-to-neck ratio less than 2
  - Too small to coil (less than 3 mm)
  - Contraindication to anti-platelet medications
  - Contraindication to stent placement (Nickel allergy)
- Complex morphology
  - Partially thrombosed
  - Previous failed endovascular treatment

## Limitations

- Aneurysm Dome Projection
  - Posterior dome projection risk factor for ischemic complications
  - Calcified aneurysm neck
- Increasing age (> 65 years increases risk of poor outcome by 11-fold)

## How to avoid complications

- Always use temporary clip (max 5 min) before permanent clipping [10].
- Identify structures that provide complications:
  1. No calcifications in aneurysm neck
  2. Thalamo-perforatorating arteries
  3. Vein of Labbe’ and temporal bridging veins
- Prefer mobilization of small veins through sharp arachnoid dissection
  1. consider sacrifice only for small, redundant veins with adequate collateral drainage
- Appropriate clip selection before starting clipping.
- Intraoperative multimodality monitoring [10].
- ICU postoperative care (different in ruptured vs unruptured cases).

## Patient information

Make the patients aware about potential complications:

- ischemic complications with brainstem or posterior circulation infarction causing coma, memory loss, or death.
- oculomotor nerve palsy (mostly temporary) from retraction, manipulation, or ischemia (30–50%) [8].
- CSF leakage with need for further treatment.
- skin- and wound-healing complications.

## Key points

1. Knowledge of complex regional and vascular anatomy
2. Understanding of aneurysm morphology and surrounding structures on imaging
3. Proper surgical approach (clival zones)
4. Maximal aneurysm exposure for proximal and distal control
5. Maximal brain relaxation
6. Maximal blood vessel visualization allows perfect clip application and vessel inspection

## Supplementary Information

Below is the link to the electronic supplementary material.

Supplementary video 1 (MP4 234 MB)

## Data Availability

Data materials are available per direct requests to the authors.

## Abbreviations

BA: Basilar artery
BAA: Basilar artery apex
SCA: Superior cerebellar artery
CTA: Computer tomography angiography
DSA: Digital subtraction angiography
ACP: Anterior clinoid process
PCP: Posterior clinoid process
PCA: Posterior cerebral artery
PTPA: Posterior thalamoperforating arteries
FTOZ: Frontotemporo-orbitozygomatic
PCoA: Posterior communicating artery
ICA: Internal carotid artery
